# Comparative analysis of clinical characteristics and outcomes between carbapenem-resistant and carbapenem-sensitive *Klebsiella pneumoniae* infections: insights from a tertiary hospital in Northern China

**DOI:** 10.3389/fmed.2025.1499057

**Published:** 2025-02-05

**Authors:** Bu Wang, Wei Zhang, Hongxia Zhang, Maochen Li, Zhihua Zhang, Xiaocui Peng, Na Wang, Ning Song

**Affiliations:** ^1^Department of Infectious Diseases, Hebei Medical University, The Second Hospital of Hebei Medical University, Shijiazhuang, Hebei Province, China; ^2^Department of Respiratory and Critical Care Medicine, The First Affiliated Hospital of Hebei North University, Hebei North University, Zhangjiakou, Hebei Province, China; ^3^Central Laboratory, The First Affiliated Hospital of Hebei North University, Zhangjiakou, Hebei Province, China; ^4^Administration Department of Nosocomial Infection, The First Affiliated Hospital of Hebei North University, Zhangjiakou, Hebei Province, China

**Keywords:** antibiotic, drug resistant bacteria, 30-day mortality, odds ratio, clinical outcomes

## Abstract

**Background:**

To compare the risk factors, clinical outcomes, and mortality rates between carbapenem-resistant *Klebsiella pneumoniae* (CRKP) and carbapenem-sensitive *Klebsiella pneumoniae* (CSKP) infections.

**Methods:**

A retrospective cohort study was conducted on patients with *Klebsiella pneumoniae* infections admitted to a tertiary hospital in Zhangjiakou, China. The research period is from January 2021 to December 2022. Data were analyzed using SPSS 24.0 and R. Univariate analysis identified potential risk factors for CRKP infections using appropriate statistical methods, followed by multivariable logistic regression to determine independent risk factors. Mortality rates between CRKP and CSKP groups were compared using chi-square tests, and survival curves were generated with the Kaplan–Meier method.

**Results:**

The study included 283 patients, with 104 (36.7%) infected by CRKP and 179 (63.3%) by CSKP. CRKP patients had significantly higher body temperature, white blood cell counts, and inflammatory markers, while showing lower diastolic blood pressure and oxygen saturation (*p* < 0.05). CRKP infections were predominantly found in the ICU (49%) and mainly isolated from sputum (59%). Independent risk factors for CRKP included elevated C-reactive protein (OR = 1.02) and solid tumors (OR = 18.186). CRKP patients experienced longer hospital stays (25 days vs. 12 days for CSKP), longer ICU stays (13 days vs. 7 days), and higher 30-day mortality (23.1% vs. 17.9%, *p* = 0.012). The deceased group had elevated procalcitonin and creatinine levels, longer prothrombin time, and a greater need for mechanical ventilation compared to survivors (*p* < 0.05).

**Conclusion:**

Patients with CRKP infections had higher mortality rates and longer hospital stays than those with CSKP infections. Previous hospitalization, hospitalization in an ICU, and mechanical ventilation were independent risk factors for CRKP infection.

## 1 Introduction

*Klebsiella pneumoniae*, a Gram-negative, non-motile, encapsulated, lactose-fermenting, facultative anaerobic, rod-shaped bacterium, is a frequent cause of hospital-acquired infections (1, 2). These bacteria, often found in the human intestines (where they do not generally cause disease), are increasingly recognized as a major global health threat due to their capacity to acquire resistance to multiple antibiotic classes (2).

Among the various resistance mechanisms, carbapenem resistance has become particularly worrisome since these antibiotics are considered last resort agents for the treatment of infections caused by multidrug-resistant Gram-negative bacteria (2–4). The enzymes responsible for carbapenem resistance (carbapenemases) are often encoded on mobile genetic elements, which enhances their potential for rapid dissemination (5, 6).

In China, carbapenem-resistant *Klebsiella pneumoniae* (CRKP) infections have become increasingly prevalent, posing significant challenges to clinical management (6–9). A teaching hospital in Shanghai found through 16 years of observational research that the mortality rate of patients infected with carbapenem-resistant bacteria is higher than that of patients infected with carbapenem-sensitive bacteria (18.8%vs.7.4%, *p* = 0.001) (10). Due to the influence of genetic factors, the ST11 KL64 CRKP high-virulence subclonal strain has a transmission advantage (11). In wastewater treatment plants in South Asian countries, carbapenemase-producing mcr-1-positive *Klebsiella pneumoniae* was detected (12). It is concerning that highly virulent and carbapenem-resistant *Klebsiella pneumoniae* has emerged globally. The emergence of these highly virulent carbapenem-resistant strains occurs mainly through three pathways: the transformation of highly virulent strains into carbapenem-resistant virulent strains; the conversion of carbapenem-resistant strains into highly virulent carbapenem-resistant strains; and the acquisition of hybrid plasmids by classic *Klebsiella pneumoniae* that carry both virulence and carbapenem resistance phenotypes (13).

Identification of the risk factors associated with CRKP infection and understanding the clinical outcomes in affected patients is crucial for effective preventative and therapeutic interventions. Previous studies have mainly focused on the molecular epidemiology and antimicrobial susceptibility of CRKP (14, 15), with less attention given to the clinical characteristics and outcomes of patients infected by these strains, particularly in Northern China. Pathogenic microorganisms exhibit regional differences in prevalence. The northern region of Hebei Province in China has a higher altitude and lower average temperatures, characterized by unique geographical features. However, there is a lack of epidemiological data on pathogenic microorganisms and the patients they cause in this area. Hence, this study aims to provide a comprehensive analysis of the risk factors and clinical outcomes of CRKP-infected patients in comparison with those infected by carbapenem-sensitive *Klebsiella pneumoniae* (CSKP) in a tertiary hospital in Northern China.

This study specifically aims to highlight the impact of CRKP infection on clinical outcomes such as duration of hospital stays, mortality rates and treatment failures. We also aim to elucidate the risk factors contributing to the acquisition of CRKP infections, which will be beneficial for developing targeted intervention and control measures.

## 2 Materials and methods

### 2.1 Study design and population

This retrospective cohort study was conducted at a tertiary hospital in Northern China, a region grappling with a surge in Carbapenem-Resistant *Klebsiella pneumoniae* (CRKP) and Carbapenem-Sensitive *Klebsiella pneumoniae* (CSKP) infections. Access patient data from January 2021 to December 2022 for research purposes.

The criteria for determining infected patients are based on authoritative guidelines and standards (16–18), with detailed standards recorded in the Supplementary materials (2 Supplementary Diagnostic Criteria for Infection). Include patients with community-acquired infections and non-community-acquired infections in the study scope.

Eligibility criteria for patients’ enrolment in the study were: (i) Age over 18 years, (ii) positive culture for *Klebsiella pneumoniae* isolated from specimens such as blood, respiratory tract, urine, wound, abdominal fluid and others, (iii) confirmation of CRKP and CSKP strains based on antimicrobial susceptibility tests in compliance with Clinical and Laboratory Standards Institute (CLSI) guidelines, (iv) patients with complete clinical records, and (v) the patient’s serological infection markers exceeded the normal range (CRP > 2.87 mg/L, or neutrophil percentage > 75%, or PCT > 0.05 ng/mL).

We excluded patients if: (i) they had polymicrobial infection, i.e., isolation of more than one type of pathogenic microorganism from the same specimen, (ii) they were transferred to other hospitals within 48 h of admission, (iii) they had recurring infections with *Klebsiella pneumoniae* within 30 days, (iv) they are non-infected patients who do not meet the infection diagnosis criteria, and (v) incomplete medical record.

Patients were grouped according to their infection type: CRKP or CSKP. Demographic clinical data, including age, gender, underlying diseases, ICU admission, invasive procedures, prior antibiotic use, infection source, severity of illness, etc., were collected from electronic health records of the patients. Collect and document all relevant laboratory data from the day the pathogen was detected. Ensure that this includes all biochemical markers, blood test results, imaging findings, and other critical laboratory data related to the patient’s condition. On the day when the bacterial culture tests positive, collect various test results to reflect the impact of the infection on the patient. This comprehensive collection will enable a thorough analysis of the patient’s clinical status.

The primary outcomes monitored included mortality (both in-hospital and 30-day all-cause), length of hospital stay, and rate of secondary complications. Ethical approval for the study was obtained per the Institutional Review Board of the hospital. All methods were performed in accordance with the relevant guidelines and regulations (19). This study involved a retrospective analysis of patient medical records. The data were accessed for research purposes on [day, month, year]. Individual participant identifiers were anonymized, and the authors did not have access to information that could identify individual participants during or after data collection.

### 2.2 Definitions of CRKP and CSKP

Automated instruments are used to identify bacteria and test their susceptibility to antimicrobial drugs (Becton, Dickinson and Company, Franklin Lake in New Jersey, United States). In this study, we define CRKP as isolates of *Klebsiella pneumoniae* that exhibit resistance to the carbapenem group of antibiotics, as determined by antimicrobial susceptibility testing. This includes resistance to any one of the following carbapenem drugs: Meropenem or Imipenem. Carbapenem resistance is identified according to the breakpoint criteria outlined in the Clinical and Laboratory Standards Institute (CLSI) guidelines (10). *Escherichia coli* ATCC 25922, and *Pseudomonas aeruginosa* ATCC 27853 were used as the quality control strain.

### 2.3 Statistical analysis

Based on information about CRKP and CSKP pathogens, access electronic medical records to obtain various test and examination data of patients, and compile them into an electronic database (raw data). The data were analyzed statistically using SPSS 24.0 software and the R Programming Language. A *p* < 0.05 was considered statistically significant. To identify potential risk factors for CRKP infections, univariate analysis was conducted using various statistical methods based on the nature of the data. Quantitative variables with a normal distribution were represented as mean ± standard deviation (S.D.) and compared using Student’s t-test. Non-normally distributed variables were expressed as median (interquartile range) [M (IQR)] and analyzed using the Wilcoxon Rank Sum Test. Qualitative data were represented as number and percentage [n (%)] and categorical variables were compared using Pearson’s Chi-square test or Fisher’s exact test with continuity correction for frequencies less than 5. Variables with a *p* < 0.05 in the univariate analysis were included in a multivariable logistic regression model. Odds ratios (ORs) and 95% confidence intervals (CIs) were calculated for each variable. In the multivariable logistic regression, risk factors with a *p* < 0.05 and OR > 1 were considered independent risk factors. The logistic regression was performed using the likelihood ratio test (forward: LR, default inclusion criteria *p* < 0.05, default exclusion criteria: *p* > 0.1). In the prognostic analysis of CRKP infection, mortality was compared between CRKP and CSKP groups using Pearson and chi-square tests, and survival curves were plotted using the Kaplan–Meier method (Log rank test).

## 3 Results

### 3.1 Physical signs and serology

**Figure 1:**
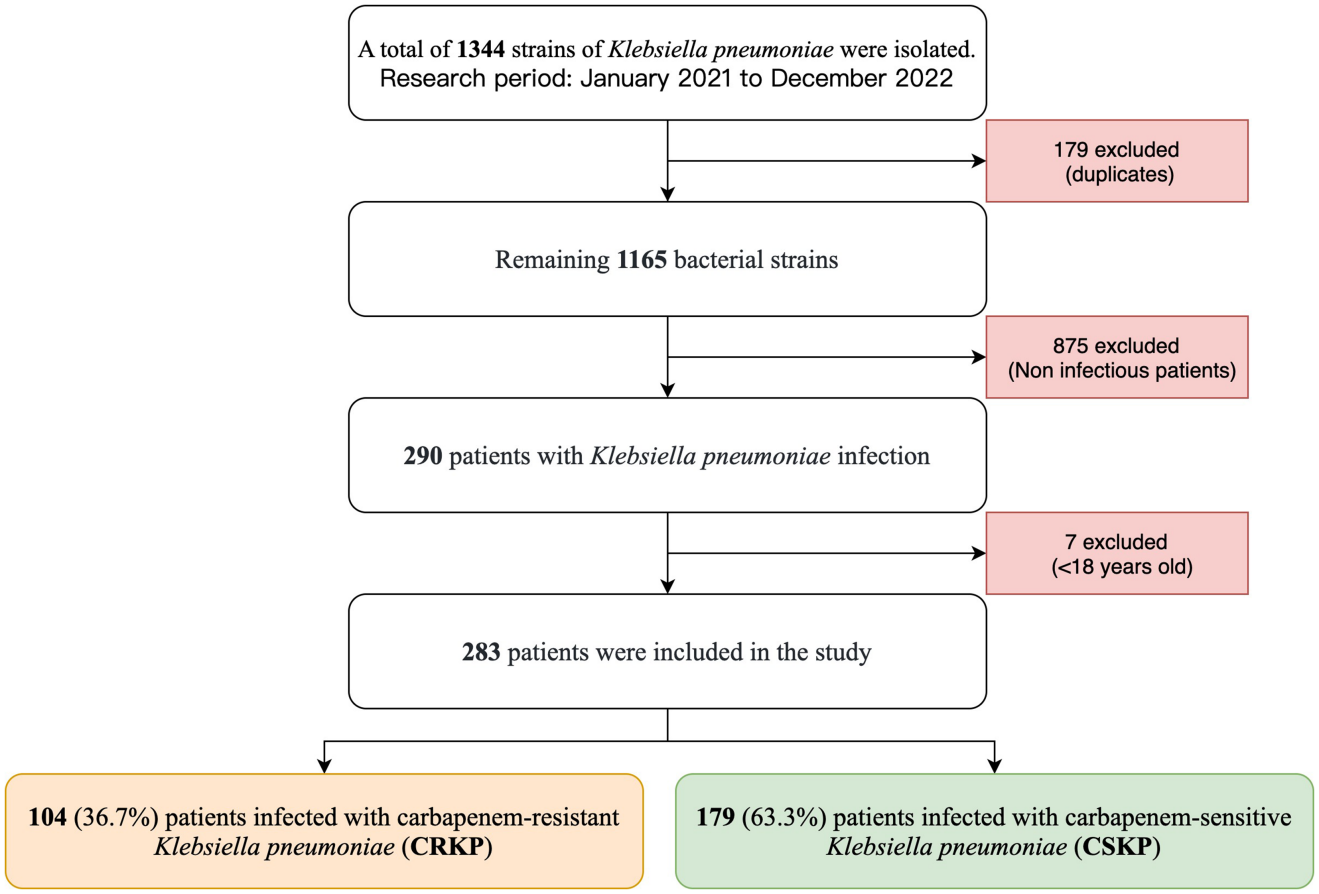
Research flowchart.

The study enrolled a total of 283 patients, excluding those who were only colonized by *K. pneumoniae* according to pre-defined exclusion criteria (Figure 1). Table 1 shows that 104 patients (36.7%) were infected with CRKP, while 179 patients (63.3%) were infected with CSKP. The CRKP infected group exhibited higher levels of body temperature, white blood cells, neutrophil percentage, procalcitonin, C-reactive protein, aspartate aminotransferase, alanine aminotransferase, creatinine, Brain natriuretic peptide, Prothrombin time, and D-dimer, while showing lower levels of diastolic blood pressure, SPO2, lymphocyte percentage, hemoglobin, Aspartate aminotransferase/Alanine aminotransferase, blood urea nitrogen, estimated glomerular filtration rate, and thrombin time (*p* < 0.05) compared to the CSKP infected group.

**Table 1:** Univariate analysis of physical signs and serological indicators in the CRKP and CSKP groups.

Variables	CRKP (*n* = 104)	CSKP (*n* = 179)	HR (95% CI)	*p* value
Vital Sign				
Systolic Pressure(mmHg)	129.5(37.25)	135(33)	6(0–13)	0.069
Diastolic blood pressure(mmHg)	76(21)	80(20)	4(0–8)	**0.042**
SpO_2_(mmHg)	90(9.25)	95(4)	3(3–5)	**0.000**
Respiratory Rate(Times/min)	20(5)	20(3)	−8.2(−1–4.5)	0.167
Heart rate(Times/min)	90(26)	83(20)	−3(−8–1)	0.169
Blood routine tests				
WBC (10^9^/L)	9.92(6.01)	7.95(5.8)	−1.5(−2.59 – −0.21)	**0.021**
ANC (%)	84.2(14.3)	77.9(24.3)	−5.9(−9.4 – −2.6)	**0.000**
LYM (%)	8.6(11.9)	16.5(19.4)	5.3(2.9–8.1)	**0.000**
PLT (10^9^/L)	176(160.8)	211(111.5)	22(−4–47)	0.098
Hb (g/L)	118(45.5)	134(35.5)	17(10–24)	**0.000**
Inflammatory parameters				
PCT (ng/mL)	0.33(1.9)	0.12(0.6)	−0.12(−0.22 – −0.05)	**0.000**
CRP (mg/L)	38.8(65.8)	10.3(28.6)	−17.2(−33.01 – −3.73)	**0.005**
IL-6(μg/L)	54.2(132.8)	40.6(117.2)	−7.1(−48.53–24.22)	0.469
ESR(mm/h)	62(71)	40(80)	−8(−31–5)	0.217
Clinical biochemical indexes				
AST(U/L)	41(49)	25(24)	−11(−19 – −4.0)	**0.001**
ALT(U/L)	32(37)	18(14)	−11(−16 – −7)	**0.000**
AST/ALT	1.3(0.98)	64.6(28.7)	63(60.2–67.3)	**0.000**
Cre(μmoI/L)	66(55.3)	6.3(3.9)	−59.5(−70.86 – −52)	**0.000**
BUN(moI/L)	7.59(9.7)	88.4(45.1)	76.2(71.17–82.69)	**0.000**
eGFR(μmoI/L)	78.4(42.7)	87(45.8)	10.4(1.94–19.38)	**0.018**
BNP(pmol/L)	121.6(264.4)	78(81.5)	−29.1(−63.25 – −2.65)	**0.029**
Tn(μg/L)	0.1(0.18)	0.06(0.15)	−0.01(−0.05 – −0.01)	0.318
Mb(ng/ml)	90.3(183.8)	76.4(249.9)	−4.4(−31.69–23.75)	0.715
K^+^(mmoI/L)	4.02(0.72)	4.03(0.65)	−0.04(−0.17–0.1)	0.624
Blood coagulation function				
PT(s)	13.3(2.5)	12.3(1.85)	−1(−1.3 – −0.6)	**0.000**
APTT(s)	29.5(7.5)	29.85(4.8)	−0.2(−1.4 – −1)	0.742
TT(s)	16.2(1.95)	17.2(1.6)	0.8(0.5–1.2)	**0.000**
FIB(g/L)	3.82(2.68)	3.79(2.59)	0.07(−0.36–0.51)	0.738
D-D(mg/L)	2.9(5.41)	0.99(1.81)	−1.44(−2.21 – −0.86)	**0.000**

Data are expressed as n (%) or median (IQR). Bold *p* < 0.05 indicates statistical significance. CRKP, carbapenem-resistant *Klebsiella pneumoniae*; CSKP carbapenem-susceptible Klebsiella pneumonia; IQR, interquartile range; HR, hazard ratio; CI, confidence interval; SpO2, Oxygen Saturation; WBC, white blood cell; ANC, neutrophil granulocyte; LYM, Lymphocyte; PLT, platelet; Hb, Hemoglobin; PCT, procalcitonin; CRP, C-reactive protein; IL-6, Interleukin-6; ESR, erythrocyte sedimentation Rate; AST, Aspartate aminotransferase; ALT, Alanine aminotransferase; Cre, creatinine; BUN, blood urea nitrogen; eGFR, estimated glomerular filtration rate; BNP, Brain natriuretic peptide; Tn,troponin; Mb, Myoglobin; PT, Prothrombin time; APTT, activated partial thromboplastin time; TT, thrombin time; FIB, Fibrinogen; D-D, d-dimer.

### 3.2 Distribution of departments and clinical samples

The majority of CRKP patients were concentrated in the intensive care unit (49%), respiratory department (12%), geriatric department (7%), and neurosurgery department (7%). CSKP patients were distributed relatively evenly across departments, with the intensive care unit (23%), geriatric department (14%), respiratory department (12%), and cardiology department (9%) being the most common (Supplementary Figure 1). The majority of CRKP isolates were obtained from sputum (59%), with urine (19%) and alveolar lavage fluid (6%) being the subsequent sources. CSKP specimens were primarily derived from sputum (75%), followed by urine (6%; Supplementary Figure 2).

### 3.3 Multivariate analysis of risk factors for CRKP infections

The majority of patients infected with *Klebsiella pneumoniae* were elderly individuals (median age 67 [IQR 19]; CRKP 66 [IQR 21] vs. CSKP 67 [IQR 18], *p* = 0.371). Male patients had a twofold higher likelihood compared to females (189 vs. 94). Over half of the patients (156/283, 55.1%) had a history of smoking. CRKP patients had a lower likelihood of comorbid Diabetes Mellitus and Hyperthyroidism (*p* < 0.05), a higher likelihood of receiving invasive medical treatments (mechanical ventilation, tracheotomy, central venous drainage; *p* < 0.05), longer duration of antibiotic use (*p* < 0.05), and a higher likelihood of using Carbapenems, third or fourth generation cephalosporins, and Quinolones (*p* < 0.05) compared to the CSKP patient group (Table 2). All variables that showed statistical significance in the univariate analysis were included in the binary logistic regression for multivariate analysis. Additionally, variables with a *p* ≤ 0.01 (hypertension, diabetes, and antifungal agents) were also included to ensure potential risk factors were not omitted. Results showed that several factors were identified as independent risk factors for CRKP infections (Table 3). These factors include C-reactive protein (OR = 1.02, *p* = 0.03), solid tumor (OR = 18.186, *p* = 0.000), cardiovascular diseases (OR = 5.689, *p* = 0.001), hypohepatia (OR = 9.843, *p* = 0.000), previous hospitalization (OR = 2.022, *p* = 0.000), hospital stay (OR = 1.098, *p* = 0.001), ICU stay (OR = 1.111, *p* = 0.005), glucocorticoids (OR = 3.340, *p* = 0.025), mechanical ventilation (OR = 2.715, *p* = 0.007), and central venous catheterization (OR = 4.043, *p* = 0.029). Some CSKP and CRKP patients received treatment with intravenous injection of methylprednisolone at a dosage of 30 mg/kg or sodium dexamethasone phosphate at a dosage of 20 mg/day.

**Table 2:** Analysis of basic diseases and treatment differences between CRKP group and CSKP group.

Variables	CRKP (*n* = 104)	CSKP (*n* = 179)	*p* value
Baseline characteristics			
^a^Age	66(21)	67(18)	0.317
^b^Male	68(65.4)	121(67.6)	0.746
^b^Smoking	64(61.5)	92(51.4)	0.353
Comorbidity			
^b^COPD (n/%)	11(10.6)	34(19.0)	0.072
^b^Coronary Heart Disease	22(21.2)	39(21.8)	0.967
^b^Hypertension	45(43.2)	79(44.1)	0.934
^b^Diabetes mellitus	21(20.2)	85(47.5)	**0.000**
^b^Hyperthyroid	1(0.96)	28(15.6)	**0.000**
^b^Cancer	2(1.92)	9(5.0)	0.176
^c^SOFA	1.8 ± 2.3	1.1 ± 1.9	**0.015**
Treatment measures			
^b^Mechanical ventilation	57(54.8)	44(24.6)	**0.000**
^b^Tracheotomy	33(31.7)	18(10.1)	**0.000**
^b^Central venous catheterization	41(39.4)	38(21.2)	**0.000**
^b^Artificial nutrition	37(35.6)	57(31.8)	0.160
Antibiotics and Hormone			
^a^Antibiotic application time	23(19)	10(11.5)	**0.000**
^b^Carbapenems	48(46.2)	24(13.4)	**0.000**
^b^Third or fourth generation cephalosporin	61(58.7)	61(34.1)	**0.001**
^b^First and second generation cephalosporins	12(11.5)	19(10.6)	0.990
^b^Penicillins and penicillins inhibitor	47(45.2)	74(41.3)	0.996
^b^Quinolones	39(37.5)	24(13.4)	**0.000**
^b^Hormone	25(24.0)	27(15.1)	0.087
^a^Hormone application time	6(3.5)	8(9)	0.382

Data are expressed as ^a^median (IQR), ^b^n (%) or ^c^mean ± SD. Bold *p* < 0.05 indicates statistical significance.

**Table 3:** Multivariate logistic regression analysis of risk factors for infections caused by CRKP group.

Variable	*p* value	OR	95% CI
Hemoglobin (g/L)	0.220	0.961	0.929–0.994
Aspartate aminotransferase (U/L)	0.367	1.004	0.996–1.011
Alanine aminotransferase (U/L)	0.405	0.996	0.986–1.006
AST/ALT	0.498	1.002	0.997–1.007
Albumin(g/L)	0.080	0.89	0.78–1.014
Lymphocyte (%)	0.162	0.954	0.892–1.019
Oxygen Saturation(mmHg)	0.001	0.869	0.802–0.941
Urea(mmol/L)	0.883	1.008	0.911–1.114
Prothrombin time (s)	0.486	1.050	0.916–1.024
Procalcitonin (ng/mL)	0.020	0.922	0.861–0.987
C-reactive protein (mg/L)	**0.003**	**1.02**	1.007–1.034
Serum creatinine (μmol/L)	0.256	1.005	0.996–1.014
white blood cell (10^9^/L)	0.493	0.970	0.891–1.057
neutrophil granulocyte (%)	0.149	0.966	0.921–1.102
Hypoalbuminemia	0.210	0.382	0.085–1.271
Solid tumor	**0.000**	**18.186**	3.881–85.212
Cardiovascular diseases	**0.001**	**5.689**	1.945–16.639
Hypohepatia	**0.000**	**9.843**	2.875–33.704
Anemia	0.019	0.107	0.017–0.689
≥three types of comorbidities	0.791	0.743	0.083–6.688
Previous hospitalization	**0.000**	**2.022**	0.781–5.225
Hospital stay (days)	**0.001**	**1.098**	1.041–1.159
ICU stay (days)	**0.005**	**1.111**	1.032–1.196
Glucocorticoids	**0.025**	**3.340**	1.166–9.968
Macrolides	0.086	1.128	0.430–0.979
Antifungal agents	0.180	0.297	0.050–1.793
Mechanical ventilation	**0.007**	**2.715**	1.307–5.641
Cheal intubation	0.063	1.420	0.279–7.233
Central venous catheterization	**0.029**	**4.043**	0.898–18.201

Bold *p* < 0.05 indicates statistical significance. CRKP, carbapenem-resistant *Klebsiella pneumoniae*; OR, odds ratio; CI, confidence interval; AST, Aspartate aminotransferase; ALT, Alanine aminotransferase.

### 3.4 Outcomes

**Figure 2:**
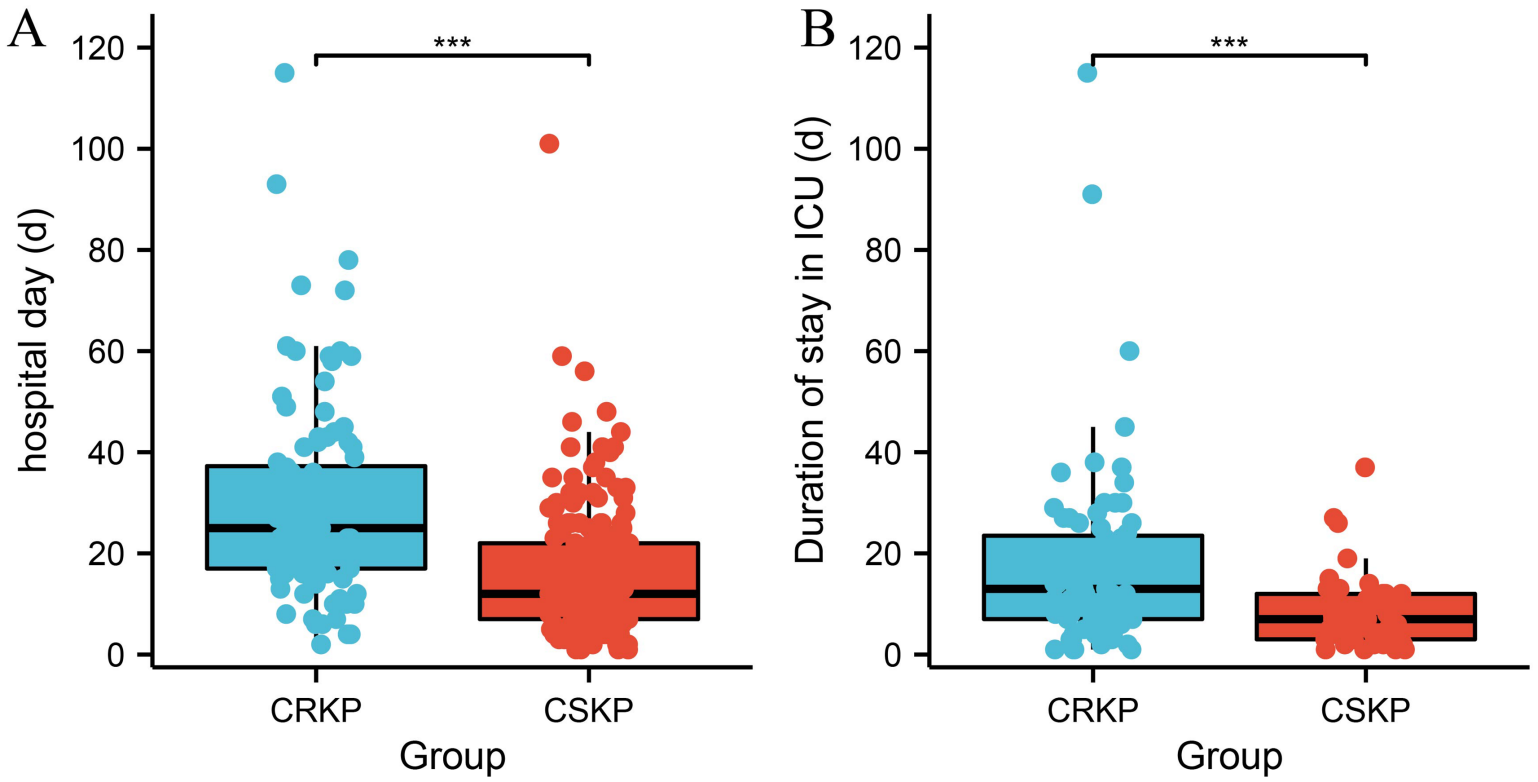
Analysis of the differences in hospitalization duration between CRKP patients and CSKP patients **(A)** and the length of ICU stay **(B)**. Carbapenem-resistant *Klebsiella pneumoniae* (CRKP), Carbapenem-sensitive *Klebsiella pneumoniae* (CSKP).

Patients infected with CRKP had significantly longer hospital stays (CRKP 25 [IQR 20.3] vs. CSKP 12 [IQR 15], *p* = 0.000), durations of stay in the ICU (CRKP 13 [IQR 16.5] vs. CSKP 7 [IQR 9], *p* = 0.000), 30-day mortality (CRKP 23.1% vs. CSKP 17.9, *p* = 0.012), and in-hospital mobility (CRKP 32.7% vs. CSKP 19.0%, *p* = 0.004) compared to patients infected with CSKP (Figure 2; Table 4).

**Table 4:** Clinical outcomes of the CRKP and CSKP groups.

Outcomes	CRKP (n/%)	CSKP (n/%)	*p* value
30-day mortality	24(23.1)	32(17.9)	0.012
In-hospital mortality	34(32.7)	34(19.0)	0.004

After classifying patients based on their outcomes (death or survival), it was discovered that the death group exhibited higher levels of procalcitonin, creatinine, troponin, and myoglobin, longer prothrombin time, a higher likelihood of mechanical ventilation, and a greater proportion of carbapenem drug usage compared to the survival group (*p* < 0.05). Additionally, the death group had lower values of lymphocyte count, hemoglobin, and estimated glomerular filtration rate (*p* < 0.05; Supplementary Table 1).

## 4 Discussion

The present study provides a comprehensive comparative analysis of risk factors and clinical outcomes between CRKP and CSKP infections in a tertiary hospital in Northern China. Our findings contribute to the growing body of literature on this critical public health issue, offering valuable insights for clinicians and researchers alike.

Bacterial colonization refers to the attachment and reproduction of bacteria on the surfaces of a host (such as skin, mucous membranes, or the intestinal tract) without triggering an immune response or symptoms in the host. In contrast, bacterial infection is the condition that occurs when pathogens enter host tissues and cause an inflammatory response. Infections are typically accompanied by symptoms such as fever, pain, and dysfunction, and can lead to more serious health issues. Therefore, accurately distinguishing between bacterial infections and colonization is crucial for clinical diagnosis and treatment. In this study, 75.1% (875/1165) of patients were excluded based on inclusion and exclusion criteria (Figure 1), as these patients may have been experiencing bacterial colonization rather than infection, thereby ensuring the scientific rigor and integrity of the research.

In this study, unlike CSKP-infected patients spread across multiple departments, CRKP-infected patients are more concentrated, with about half of them coming from the ICU (Supplementary Figure 1). Correspondingly, CRKP is more widely distributed in samples compared to CSKP, this study focuses primarily on patients with pneumonia, categorized into community-acquired pneumonia, hospital-acquired pneumonia, and ventilator-associated pneumonia. Although these three types of pneumonia are all pulmonary infections, there are significant differences in their occurrence environments, pathogen characteristics, clinical manifestations of patients, and management approaches (Supplementary Figure 2). Recent admission to the ICU and the use of carbapenem antibiotics are independent risk factors for CRKP infection (20). *Klebsiella pneumoniae* of different genotypes colonize in plants, animals, and humans, and can be transmitted through contaminated water, causing community-acquired and hospital-acquired infections in human populations (21). In ICU patients, the use of carbapenem antibiotics, tigecycline, or β-lactam/β-lactamase inhibitors, as well as undergoing invasive procedures or surgery, are considered risk factors associated with CRKP colonization (22, 23). Differentiating the causal relationship between ICU hospitalization and CRKP infection outcomes is a complex task. This challenge can be addressed through strategies such as prospective cohort studies, adjustment for confounding variables, propensity score matching, case–control studies, and long-term follow-up. The retrospective design of this study limits the ability to make clear causal inferences. This study used serum infection markers (CRP, PCT, NEUT%) to screen for patients infected with *Klebsiella pneumoniae*, avoiding bias caused by bacterial colonization. In patients with CSKP infection, the levels of CRP, PCT, and IL-6 in the death group were significantly higher than those in the survival group (*p* < 0.05), indicating that the values of these three indicators are positively correlated with the severity of the infection (Supplementary Table 1). That is, the higher the levels of CRP, PCT, and IL-6, the worse the prognosis for patients with CSKP infection. In comparison to the survival group, patients who succumbed to CRKP infection had higher levels of PCT, CRP, and NEU% (23). Compared to patients with CSKP infections, those with CRKP infections exhibited more pronounced clinical symptoms and significant changes in biochemical markers. Specifically, CRKP-infected patients had notably higher levels of body temperature, white blood cell count, procalcitonin (PCT), C-reactive protein (CRP), aspartate aminotransferase (AST), alanine aminotransferase (ALT), creatinine (Cre), prothrombin time (PT), and D-dimer (D-D; *p* < 0.05), indicating a more severe inflammatory response and organ damage.

Meanwhile, CRKP-infected patients showed significantly lower levels of diastolic blood pressure, partial pressure of oxygen (PaO2), lymphocyte count, hemoglobin levels, blood urea nitrogen (BUN), estimated glomerular filtration rate (eGFR), brain natriuretic peptide (BNP), and thrombin time (TT) compared to those with CSKP infections (*p* < 0.05). The decline in these indicators may be associated with more severe systemic circulatory disorders, reduced oxygenation capacity, and suppressed immune function.

Additionally, CRKP-infected patients underwent more invasive medical procedures, including mechanical ventilation, tracheostomy, and central venous catheterization (*p* < 0.05), which likely reflects the complexity and severity of their condition. The frequency of antibiotic use was also significantly higher in this group, with increased usage of carbapenems, third-generation cephalosporins, and quinolones, as well as a longer duration of antibiotic therapy (*p* < 0.05). Patients colonized with CRKP have a higher exposure rate to carbapenems and third-generation cephalosporins compared to non-colonized patients (22). Notably, the prevalence of diabetes and hypertension was lower among CRKP-infected patients (*p* < 0.05), suggesting potential differences in the distribution of this infection among various populations with underlying conditions. Compared to CRKP, diabetic patients are more susceptible to infections caused by CSKP (Table 2). The potential mechanisms for CSKP infection in diabetic patients mainly include several factors such as decreased immune function, hyperglycemic environment, microvascular complications, chronic inflammatory state, imbalance of gut microbiota, effects of comorbidities, history of antibiotic use, and poor wound healing ability. These mechanisms collectively increase the risk of CSKP infection in diabetic patients.

Our study identified several risk factors associated with CRKP infections, consistent with previous research conducted both domestically and internationally. These risk factors underscore the complexity of managing CRKP infections and highlight the need for a multifaceted approach to infection control (8, 22, 24–29). However, it is worth noting that the prevalence and significance of these risk factors may vary across different geographical regions and healthcare settings due to differences in antibiotic usage patterns, infection control practices, and patient populations.

In terms of clinical outcomes, our study found that patients with CRKP infections had worse outcomes compared to those with CSKP infections. In this study, patients with CRKP infections had significantly longer hospital stays and extended ICU durations compared to those with CSKP infections (*p* < 0.05; Figure 1). Additionally, both the in-hospital mortality rate and the 30-day mortality rate were notably higher in the CRKP group (*p* < 0.05; Table 4). This aligns with numerous studies worldwide that have reported higher morbidity and mortality rates among patients with CRKP infections (25, 30–33). Statistics show that the reported mortality rates for CRKP-infected patients are 33.24% in North America, 46.71% in South America, 50.06% in Europe, and 44.82% in Asia (33). These findings underscore the increased severity and poorer prognosis associated with CRKP infections. However, further research is needed to elucidate the mechanisms underlying these poorer outcomes and to develop effective strategies to improve patient prognosis.

## 5 Conclusion

In conclusion, our study underscores the urgent need for continued research on CRKP infections. Future studies should aim to validate our findings in different settings and explore innovative strategies for preventing and managing these infections. It is essential to conduct broader population-based research to better understand the epidemiology of CRKP infections and to carry out intervention trials aimed at evaluating effective treatment strategies and preventive measures. Despite the challenges, we believe that through concerted efforts, we can make significant strides in combating this global health threat.

## Data Availability

The original contributions presented in the study are included in the article/Supplementary material, further inquiries can be directed to the corresponding author.
